# Sensing Framework for the Internet of Actors in the Value Co-Creation Process with a Beacon-Attachable Indoor Positioning System

**DOI:** 10.3390/s21010083

**Published:** 2020-12-25

**Authors:** Keiichi Zempo, Taiga Arai, Takuya Aoki, Yukihiko Okada

**Affiliations:** 1 Faculty of Engineering, Information and Systems, University of Tsukuba, Tsukuba 305-8573, Ibaraki, Japan; okayu@sk.tsukuba.ac.jp; 2 Graduate School of Systems and Information Engineering, University of Tsukuba, Tsukuba 305-8573, Ibaraki, Japan; arai@aclab.esys.tsukuba.ac.jp (T.A.); aoki@aclab.esys.tsukuba.ac.jp (T.A.)

**Keywords:** indoor positioning, navigation, storehouse, attachable beacon, infrared communication, sensor network

## Abstract

To evaluate and improve the value of a service, it is important to measure not only the outcomes, but also the process of the service. Value co-creation (VCC) is not limited to outcomes, especially in interpersonal services based on interactions between actors. In this paper, a sensing framework for a VCC process in retail stores is proposed by improving an environment recognition based indoor positioning system with high positioning performance in a metal shelf environment. The conventional indoor positioning systems use radio waves; therefore, errors are caused by reflection, absorption, and interference from metal shelves. An improvement in positioning performance was achieved in the proposed method by using an IR (infrared) slit and IR light, which avoids such errors. The system was designed to recognize many and unspecified people based on the environment recognition method that the receivers had installed, in the service environment. In addition, sensor networking was also conducted by adding a function to transmit payload and identification simultaneously to the beacons that were attached to positioning objects. The effectiveness of the proposed method was verified by installing it not only in an experimental environment with ideal conditions, but posteriorly, the system was tested in real conditions, in a retail store. In our experimental setup, in a comparison with equal element numbers, positioning identification was possible within an error of 96.2 mm in a static environment in contrast to the radio wave based method where an average positioning error of approximately 648 mm was measured using the radio wave based method (Bluetooth low-energy fingerprinting technique). Moreover, when multiple beacons were used simultaneously in our system within the measurement range of one receiver, the appropriate setting of the pulse interval and jitter rate was implemented by simulation. Additionally, it was confirmed that, in a real scenario, it is possible to measure the changes in movement and positional relationships between people. This result shows the feasibility of measuring and evaluating the VCC process in retail stores, although it was difficult to measure the interaction between actors.

## 1. Introduction

**Figure 1:**
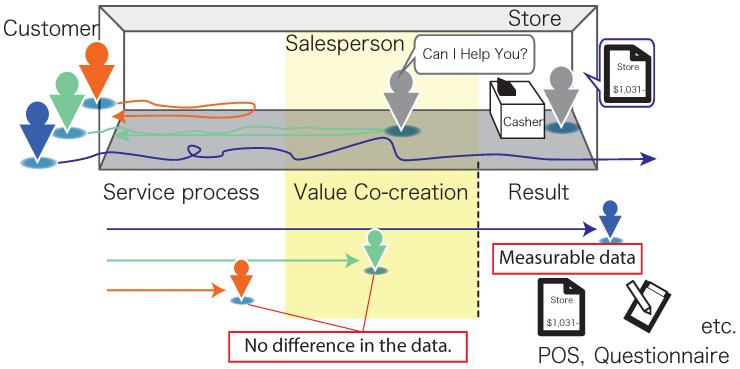
Value co-creation process of services that should be measured, which has been overlooked, considering customer service in retail stores as an example.

To evaluate and improve the value of a service, it is important to measure not only the outcomes, but also the process of the service. Value co-creation (VCC) is not limited to outcomes, especially in interpersonal services based on interactions between actors [1,2,3,4,5]. In production management or mere sales, there may be no problems with the resulting productivity or sales data; for example, in the retail industry, involving customer service, it is possible to evaluate the value of the VCC process based only on the interaction between customers and stores and between customers and salespeople (Figure 1).

Customer behavior in stores plays a significant role in determining service value, and the boundary between companies and customers has become obscure in recent years. It is necessary to measure customer engagement behavior in VCC in multi-stakeholder service systems [6,7]. In the VCC process, which includes customer service and reception in retail stores, interaction information such as the positional relation and conversation content is important. As an example of positioning service, the Global Navigation Satellite System (GNSS; including Global Positioning System (GPS)) technology is used to position a moving vehicle in an automatic driving service such as Google car [8]. In addition, the position of both parties is determined in a delivery service in which individual drivers exist, such as Uber Eats [9]. The current GPS positions of mobile devices are used to match the receiver and deliverer to enable the delivery of goods between individuals. GPS technology is also used in mobile games using augmented reality such as Pokémon GO, which links the real position coordinates of the player with the content of the Pokémon game [10].

However, positioning becomes impossible in tunnels and indoor environments. GNSS technology performs positioning by receiving radio waves transmitted from an artificial satellite, but radio waves from the satellite are shielded by the walls and roofs of tunnels and buildings. Indoor positioning technology is required when GNSS positioning is unavailable. The indoor positioning technique is still being extensively studied through various techniques [11]. This is because several indoor positioning technologies require the use of transmitters, receivers, and computers to position environments and objects. Although various indoor positioning techniques are being used practically for each application, it is difficult to use them comprehensively; each technique has merits and demerits, and the error factors are different in each technique. For the purpose of measuring VCC process, there is no specific method suitable for a service environment with many and unspecified objects. In addition, it is difficult to install a smartphone in all measured objects in positioning using a mobile terminal, which is considered to be versatile, such as a smartphone. For example, in the case of a positioning method to track an object to be measured by using a camera, it is possible to position an unspecified number of objects; however, a computer with large computational power is required for further image processing. In addition, when the targeted object is a human, the consideration of privacy anonymization is necessary. Although there are certain methods used to measure a discrete area in which a customer is present using radio frequency identification (RFID) or analyze it using a monitoring camera, it is difficult to measure continuous interaction, and it is possible to measure only sensitive and positional information.

**Figure 2:**
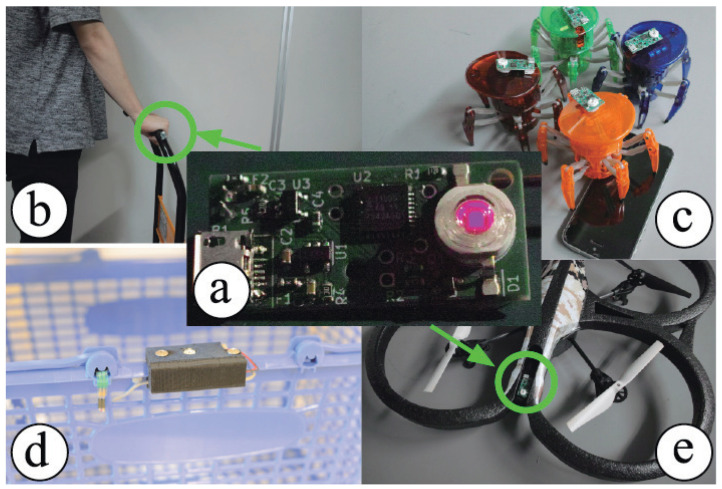
(**a**) Infrared (IR) beacon and applications; (**b**) hand cart; (**c**) small robots; (**d**) shopping basket; and (**e**) multicopters.

In this study, to measure the interaction between customers and stores or between customers and salespeople, we propose an environmental-recognition based indoor positioning system (infrared beacon localization system (IRBL)) using infrared (IR) rays in which the devices to be attached to measurement objects are small and are less affected by metal shelves, as shown in Figure 2. IR light, which is utilized to send the information containing the ID of a sender device attached on a targeted object, is measured by a receiver to obtain its direction of arrival; consequently, the receiver can simultaneously measure the positions of objects in large numbers. We reported some of these research results in [12,13].

In this paper, we first describe the basic principle and design of the positioning system and then present a comparison of the positioning accuracy in the metal shelf environment between the proposed method and the Bluetooth Low-Energy (BLE) method, which is widely used as a conventional method. Moreover, the movement of humans is measured in an environment in which the effect of metal shelving is significant, such as a retail store. In addition, this paper performs a basic examination in anticipation of the simultaneous recognition of several elements and sensor networking. Through the above, we aim to realize an Internet of Actors (IoA) that can monitor not only location information, but also the interaction among service actors that compose the services, including customers, salesperson, products, and other objects, in the service environment.

## 2. Related Works

There are various indoor positioning techniques that are characterized by their recognition type, communication system, signals used for positioning, calculation techniques, etc. Table 1 enumerates the major positioning methods classified by these features.

Recognition methods are predominantly classified into environment recognition and self-recognition methods [26,27]. Self-recognition methods include techniques used to estimate the relative position to the initial position of a measured object by attaching a transmitter, a receiver, and a computer. This method requires a device with a sensor and computational ability such as a smartphone, which is widely used predominantly in navigational applications, in which it is used as an interface [28,29].

The environment recognition method is used to estimate the position of a measured object within the range of the positioning environment by introducing a transmitter, a receiver, and a computer. There are few functions that must be given to the measurement target, and this method has the advantage that installation is easy even if there are many measurement targets. Conversely, when the positioning environment changes, it is necessary to adjust the attached equipment again. In particular, indoor positioning using the environment recognition method can track a large number of measurement objects because computational ability is not required for the equipment to be installed on a measurement object, thus incurring a low cost. For example, because anti-theft RFID tags are inexpensive, they can be attached to a large number of goods [30]. In recent years, the analysis of camera images has advanced, and the position of a person can be obtained from these images [31].

Communication systems are divided into active and passive types [32]. The active type is a positioning technique in which a system that computes a position actively transmits a signal necessary for positioning; moreover, in certain cases, the device attached to a positioning object is required to not only respond to the sender, but also to perform some calculations to obtain positions. Although there is strong merit of this technique in its robustness against disturbance, when the positioning object is a human, there are complications such as the starting of a device and the application for positioning. Thus, when the attachment of the device to the positioning object becomes complicated and the number of positioning objects is unspecified and large, it can be said that the introduction of the positioning environment and the manufacturing cost of the device represent disadvantages of this method. The passive method is a positioning technique in which a system computing a position does not transmit a signal. Therefore, when a positioning object is a human, processing such as starting a device or an application is unnecessary, and the positioning can be subconsciously performed. Moreover, the device attached to the positioning object can be simple and inexpensive for mass production, allowing a large number of positioning objects to be positioned.

The positioning method is also classified based on the signal used for positioning. Examples include the utilization of radio waves such as Wi-Fi and BLE, the visible light emitted by lighting equipment installed in indoor environments, sound waves such as ultrasonic waves, the angular velocity obtained using an acceleration sensor attached to the measurement object, odometry if the object moves by wheels, and image data [11]. When radio waves are used, a Wi-Fi system is often installed in indoor environments, and there is the advantage that using this technique without the positioning of an existing setup (such as a network infrastructure, which a Wi-Fi system originally is) is easy [33,34]. BLE has the advantage of being implemented as a smartphone application because a beacon, which acts as a transmitter, can be installed in the environment and a smartphone can be used as a receiver and attached to a positioning object. However, it has the disadvantage of being easily affected by interference, reflection, and diffraction by metals [16]. When visible or IR light is used, it is not affected by metals when compared to the case of using radio waves; however, there is a disadvantage that it is necessary to consider the effect of the shielding of light, and the problem of the disturbance of the light exists in the environment [35,36,37,38]. In the case of sound waves, installation in the environment is relatively easy; however, when multiple sounds are present, it is necessary to consider the effects of the superposition of sound waves and environmental sound [39,40,41]. When positioning is performed based on angular velocity or odometry, there is almost no requirement for a device to be introduced into the positioning environment, because only the movement of the moving object is measured. However, in several cases, changes in position coordinates are calculated by integration and anchoring, which result in measurement errors due to integration errors, making this approach unsuitable for long-term measurement [42,43]. When using image data, it is unnecessary to attach a measuring instrument to the object for measurement; moreover, it is possible to perform continuous measurement even when there are multiple objects to be measured. However, not only does the calculation cost increase, but also if the object is a human, the face of the person might be recorded; consequently, privacy anonymization is required [21,44].

A positioning method is also classified based on the calculation methods of an object’s position [45]. For example, in geometric calculation methods such as triangulation and trilateration, the relative position of a measurement object is estimated from the geometric relationship between known points and sensor values obtained by measurement equipment [46,47,48,49,50]. Therefore, even if positional relations of objects in the positioning environment change, if the positional relation of the measuring instrument does not change, it is possible to perform positioning as it does not require prior learning; moreover, the instrument can easily be attached in the environment. However, the positioning accuracy varies depending on individual differences in measuring instruments. When the position of the positioning object is calculated based on data matching, the sensor values obtained for each position pair of a transmitter and receiver are required to be measured beforehand and to be stored in a database. Then, the position is estimated by calculating the relative position by comparing the sensor value obtained in actual positioning with the value of the stored data [51,52,53,54,55,56,57,58,59]. As the positioning is performed by aggregating the data once, there is no effect on the positioning accuracy even if there is an individual difference in the measuring instruments; however, it can be said that the installation cost to the environment increases. This is because the data must be collected again when the positional relation of the goods and environmental objects in the positioning environment changes. When the position of the positioning object is calculated based on integration and anchoring, the positioning is performed by the double integration of data from an acceleration sensor or angular velocity sensor mounted on the positioning object [60,61,62,63,64,65]. However, there is no requirement for installing the equipment in the positioning environment, and the installation is comparatively simple; however, this method is not suitable for positioning over a long period of time because the integral error increases as the measurement period increases. Therefore, in the case of active self-positioning, a model that performs estimation by a combination of the integral of acceleration and RSSI (received signal strength indicator) based positioning by communication between target devices with a Kalman filter has also been developed [66]. However, this requires sufficient sensors and computing power for the positioning target.

The IRBL indoor positioning method proposed in this paper is an environment-recognition based, passive, and geometric calculation based method using IR light. This method results in an improvement in accuracy by its integration with other positioning methods; it is expected to perform in an environment surrounded by metal shelves such as retail stores, factories, and distribution warehouses. Thus, when we consider the measurement of customers and salespeople in retail stores, workers in factories and distribution warehouses, and working vehicles, a passive-type method is appropriate because the measured subjects are many and unspecified, and the hardware cost, installation cost, and calculation cost are low. Moreover, it is desirable to use IR light as it is not affected by a metal shelf or the moisture contained in the human body and is not visible to humans. Furthermore, since these environments are often changed by refurbishment, such as moving shelves and making modifications to the facilities, there is an advantage of a geometric method that does not require prior learning for position calculation. A similar technique was used to measure the angle of arrival (AoA) of signals using an IR phototransistor array to achieve indoor positioning in [25]. In the research, indoor positioning was realized by measuring the AoA of signals emitted from IR beacons mounted on shopping carts in supermarkets. This clearly showed that, compared to other methods, methods such as IRBL are most suitable for measuring human flow in an indoor space such as a store and are also superior to other methods for their combination and complementation. However, in order to realize an IoA that measures the interaction between service actors (customers, salesperson, products, and other objects) and clarifies the VCC process, which is the purpose of this research, positional information alone is insufficient. In the method proposed in this study, the function of a sensor network is considered as a development of the research in this field and a feasibility study is carried out. Based on the above, the VCC process is measured to realize an IoA.

## 3. Principal of Positioning System Based on the Angle of Arrival

### 3.1. Overview

**Figure 3:**
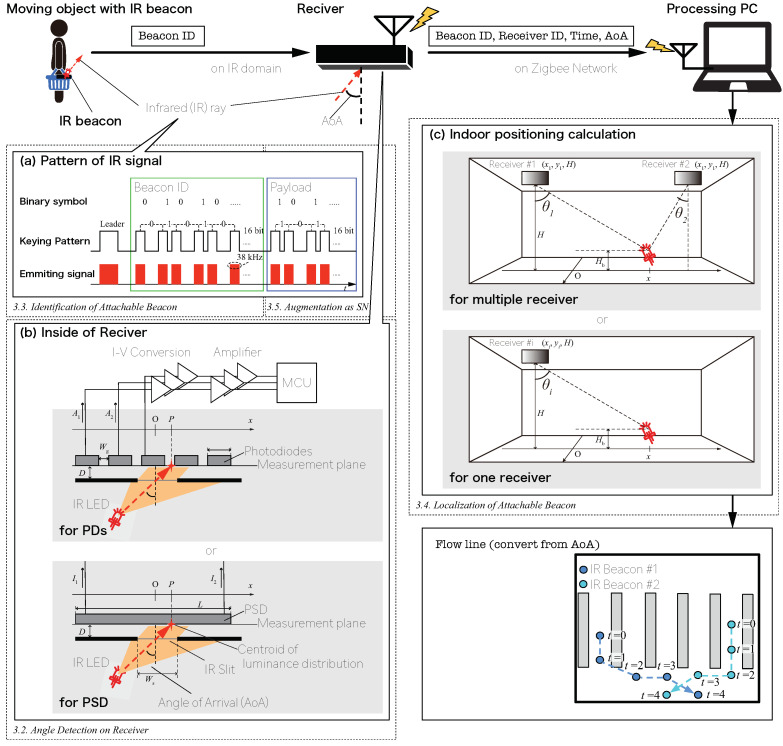
Architecture of the indoor positioning system. This positioning system is composed of beacons, receivers, and the processing PC. The IR beacon sends ID signals and is attached to objects. The receivers are installed on the ceiling. They demodulate the IR signal into information that the processing PC uses to construct the movement map. (**a**) Example of the emission pattern of the IR LED modulated by pulse-width modulation and the method for inserting the payload into the IR signal; (**b**) Mechanism of the slit “spotlights”, a part of the measurement plane; (**c**) Indoor positioning method for performing positioning with multiple angles of arrival, i.e., $\theta_{1}$ and $\theta_{2}$, and when a signal is received by only one receiver. PSD, position-sensitive detector.

The outline of the proposed positioning system is shown in Figure 3. The system consists of IR beacons attached to the measurement object, IR receivers attached to the environment, and an aggregation server to collect the signals of the receiver. The IR beacon emits IR light at intervals, and the light is measured by the receiver. The ID signal is modulated in the IR light, and different fluctuations in the interval enable simultaneous positioning for multiple beacons. The receiver obtains the sensor value based on the luminance distribution on the light-receiving surface corresponding to each incoming direction by receiving the IR signal emitted from each IR beacon through the slit by the diode array and estimates the incoming direction (angle of arrival (AoA)). By three-dimensionally matching the arrival directions of IR beacons detected by multiple receivers, the environment side calculates the measurement positions of the positioning objects.

### 3.2. Angle Detection on the Receiver

The angle measurement unit measures the angle of incidence of a signal emitted from a beacon by using a slit and a sensor (e.g., arrayed photodiodes (PDs) and a position-sensitive detector (PSD)).

The slit “spotlights” a part of the sensor, generating a unique luminance distribution on the sensor surface corresponding to the angle of incidence. The slit also has a spectral filter to remove noises. In this paper, IR-96 (Fujifilm) is used as the spectral filter. AoA measurement is achieved by utilizing the luminance distribution; in particular its centroid. For example, in case PDs arrayed on a plane are used, as the sensor is shown in Figure 3b, when two adjacent PDs are irradiated, the PDs output and the centroid of luminance distribution have the following relationship:

(1) $$A_{1}:A_{2}=\left(\frac{1}{2}\left(W_{s}-W_{g}\right)-P\right):\left(\frac{1}{2}\left(W_{s}-W_{g}\right)+P\right)\;,$$

where $A_{1}$ and $A_{2}$ are the current flowing out from each PD, $W_{s}$ is the longitudinal size of the slit, $W_{g}$ is the gap between two PDs, and *P* denotes the centroid of the luminance distribution. *P* is arranged as follows:

(2) $$P=\frac{W_{s}-W_{g}}{2}\cdot \frac{A_{2}-A_{1}}{A_{1}+A_{2}}\;.$$

In the case of PSD, AoA measurement is achieved in a similar manner, as follows:

(3) $$I_{1}:I_{2}=\left(\frac{1}{2}L-P\right):\left(\frac{1}{2}L+P\right)\;,$$

where $I_{1}$ and $I_{2}$ are the current flowing out from output terminals of the PSD and *L* is the effective length of the PSD. *P* is calculated as:

(4) $$P=\frac{L}{2}\cdot \frac{I_{2}-I_{1}}{I_{1}+I_{2}}\;.$$

Finally, the incident angle θ is obtained as:

(5) $$\theta =\tan^{-1}\frac{P}{D}\;,$$

where *D* is the distance between the sensor surface and the slit.

The maximum angular view $\theta_{\max}$, which is half of the angle of view, is determined as:

(6) $$\theta_{\max}=\tan^{-1}\frac{L-W_{s}}{2D}\;.$$

In this paper, D=6mm, $W_{s}=5\;\mathrm{mm}$, and the PSD of S3270 (Hamamatsu Photonics, L=37mm) is used, resulting in a designed $\theta_{\max}$ of approximately 69.4deg.

Because this method is basically used to measure the two-dimensional position of a beacon inside of a circular sector towards the front of a receiver, it is necessary to avoid receiving IR light from off-the-line positions. Therefore, the lateral size of the slit should be the minimum possible to cover the measurement area. This treatment would also reduce the effect of multipath, as most of the reflectors surrounding the measurement area (e.g., products displayed on metal shelves) would then be out of sight.

On the other hand, true three-dimensional positioning can be also achieved by placing two sensors orthogonally. In this case, the lateral size of the slit should be widened to cover the measurement area. However, the positioning might become less tolerant of reflection. Note that the discussion above only refers to a planar sensor surface for simplicity, but it is not a requirement.

The receiver also has the function of reading a beacon ID and the payload embedded in the signal. After AoA measurement, the receiver sends a report composed of the measured AoA, the beacon ID, the receiver ID, and the payload to the processing PC via the ZigBee network; the localization process is described below.

### 3.3. Identification of the Attachable Beacon

Because the IR beacon is assumed to be simply attached to the position measurement object, it is designed through inexpensive manufacturing processes and includes few processing abilities. It is composed of an IR light-emitting diode (LED), an ARM Cortex-M0 microcontroller, and a lithium polymer battery.

The main function of the beacon is to send information containing its unique ID and small payload to a receiver by blinking an IR LED, allowing the receiver to identify and localize the beacon. The payload can be utilized with some values obtained from the beacon’s sensor, which is described below.

In this paper, the emitting signal pattern is described as follows. A carrier wave is modulated by on–off keying, representing a leader symbol followed by information. The leader symbol, the binary “0” symbol and the binary “1” symbol are represented as different keying patterns: the leader symbol consists of ON for $16t_{p}$ and OFF for $8t_{p}$; the binary “0” symbol consists of ON for $t_{p}$ and OFF for $t_{p}$; and the binary “1” symbol consists of ON for $t_{p}$ and OFF for $3t_{p}$, where the pulse unit length $t_{p}$ is 562 µs. The amount of information is 32bits including a 16bit beacon ID and 16bit additional payload (described below). The mean signal length becomes $120t_{p}=67.44\;\mathrm{ms}$ under these parameters. A 38 kHz square wave is used as a carrier to avoid interference from noise, which is usually at a low frequency. An example of the signal is shown in Figure 3a. By emitting such a signal periodically, the position of the beacon is continuously localized by the receiver.

The transmission interval can be adjusted depending on the utilization condition. When multiple IR beacons exist within the coverage of one receiver, signal conflicts occur. After a transmission becomes conflicted, the following transmissions are also conflicted, if a constant transmission interval is used. Therefore, the transmission interval $T_{i}$ must have some jitter to avoid consecutive missing locations:

(7) $$\begin{array}{ccc} T_{i} & = & T+\tau \;, \end{array}$$

(8) $$\begin{array}{ccc} \tau & ∼ & U(-JT,JT), \end{array}$$

where *T* is a mean transmission interval, U(a,b) is a continuous uniform distribution in [a,b], and J∈[0,1) is a fluctuation ratio to *T*. Here, there is a trade-off relation between the number of beacons and the update frequency, and positioning is possible while avoiding temporal conflicts by reducing the number of beacons when frequent updates are desired or by reducing the update frequency when the number of beacons is an important factor.

### 3.4. Localization of the Attachable Beacon

One or more AoA reports about one transmission from a certain beacon would be received by the processing PC as the receivers’ coverages overlap with each other. The localization is performed on the PC by combining the reports.

Figure 3c shows how to localize the beacon when multiple receivers are receiving IR signals. As the absolute position of the receiver is known, the beacon can be localized three-dimensionally using two or more AoAs measured by the receivers. The beacon position (x,z) is obtained by solving the following equations simultaneously:

(9) $$x+z\tan\theta =H\tan\theta +x_{i}\;\left(1\leq i\leq N\right)\;,$$

where *H* is the pre-measured receiver height, *N* is the number of receivers that receive the signal from the beacon, $x_{i}$ is the pre-measured x-axis position of the *i*-th receiver, and $\theta_{i}$ is the AoA obtained by the *i*-th receiver. Finally, by combining $y_{i}$, the y-axis position of the *i*-th receiver, the three-dimensional beacon position $\left(x,y_{i},z\right)$ is obtained. If (9) is overdetermined (N≥3), it should be solved using the least squares method in order to minimize the localization error. Conversely, if (9) is underdetermined (N=1), the reasonable assumption discussed below might be helpful in the case of incomplete measurement.

While this method can localize the beacon horizontally and vertically, each position in a measurement area must be covered by at least two receivers, increasing the number of receivers. However, by introducing the assumption that the beacon height is constant (e.g., the beacon is installed on a shopping cart is moving on a flat floor), it is possible to conduct localization with some accuracy even when the signal is received by a single receiver, as shown in Figure 3c. Under this assumption, the position *x* is obtained by solving the following equations simultaneously:

(10) $$x=\left(H-H_{b}\right)\tan\theta_{i}+x_{i}\;\left(1\leq i\leq N\right),$$

where $H_{b}$ is the pre-measured beacon height. If (10) is overdetermined (N≥2), it should be solved in a similar manner as (9). Utilizing this assumption even in the case of multiple receivers being available may be helpful to achieve higher accuracy.

Since these are AoA based triangulation methods, there is a trade-off between the coverage and the accuracy of measurement; the localization error becomes greater as *H* becomes larger.

### 3.5. Augmentation as a Sensor Network

As described in Section 3.3, the system can perform unidirectional communication to send an additional payload, as well as the beacon ID; therefore, it is possible to use the communication to form a sensor network. For example, when an IR beacon is attached to a shopping basket, the total weight of goods in the basket can be sensed and transmitted by attaching a strain gauge to the basket.

Therefore, the proposed system can be extended to a sensor network to gather the sensor data. Figure 3a illustrates how the beacon ID and the sensor data are packed into the signal. The additional payload follows the beacon ID. In this paper, the microcontroller installed in the IR beacon is programmed to send the 16 bit sensor data obtained from a strain gauge as the payload.

It can, of course, also be programmed to measure and send arbitrary sensor data, such as luminance, noise level, odor, and heartbeat rate. However, the mean signal length increases as the payload size increases, meaning that the possibility of collision with other beacons increases; therefore, the payload size should be determined according to the specifications required for applications. These are discussed in Section 5.4.

## 4. Indoor Positioning Experiment in an Environment Surrounded by Metal Shelves

### 4.1. Overview

**Figure 4:**
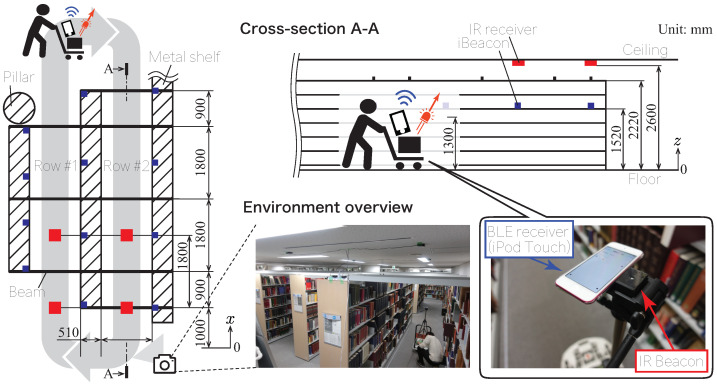
Passage environment surrounded by metal shelves. An IR beacon and Bluetooth Low-Energy (BLE) receiver (iPhone operating system based device) were placed on the cart, as a mobile object, to perform positioning simultaneously.

To evaluate the positioning accuracy of the proposed system when compared to the existing method, a positioning experiment was performed in a passage-type environment surrounded by metal shelves, as shown in Figure 4. This positioning experiment was performed with the assumption of the following two situations regarding the movement of the object.

Case 1 The object continues to be stationary in the positioning environment (positioning in a static environment);
Case 2 The object continues to move in a positioning environment (positioning in a dynamic environment).

Case 1 is an indoor positioning system that uses an IR beacon and BLE when there is only one positioning object; moreover, positioning using a commercialized BLE beacon (iBeacon, Apple Inc., Cupertino, CA, USA) was performed as a benchmark. This indoor positioning system based on the iBeacon has an iPhone operating system based device attached that receives the radio wave from the iBeacon, which is transmitted through a Bluetooth device installed in the environment to the positioning object, which acts as a receiver [67,68]. This indoor positioning system uses the fingerprinting method, which estimates the position coordinates of the positioning object by comparing the value of the reception strength (received signal strength indicator (RSSI)) as in the case of the conventional system using BLE [69,70,71]. When the receiver receives the surrounding Bluetooth radio waves, it will self-localize with the data sets of the received strength associated with the position coordinates in the environment in advance. In addition, as the IR beacon is supposed to be used even when multiple IR beacons exist in the same environment, positioning is performed in this situation. As comparisons for indoor positioning, RFID and Wi-Fi signals can be considered as methods that use the same radio waves. However, these were excluded; they are not suitable for use at the mobile scale of shoppers, which is the subject of this research, because the former is a spot-detection technique. Additionally, it is not appropriate to increase the number of access points to improve accuracy because the latter is a positioning method that uses the existing infrastructure. In Case 1, the measurement accuracy was calculated for performance evaluation, and in Case 2, an experiment was conducted to verify the consistency of the measured movement as a reference for actual use.

### 4.2. Positioning in a Static Environment

**Figure 5:**
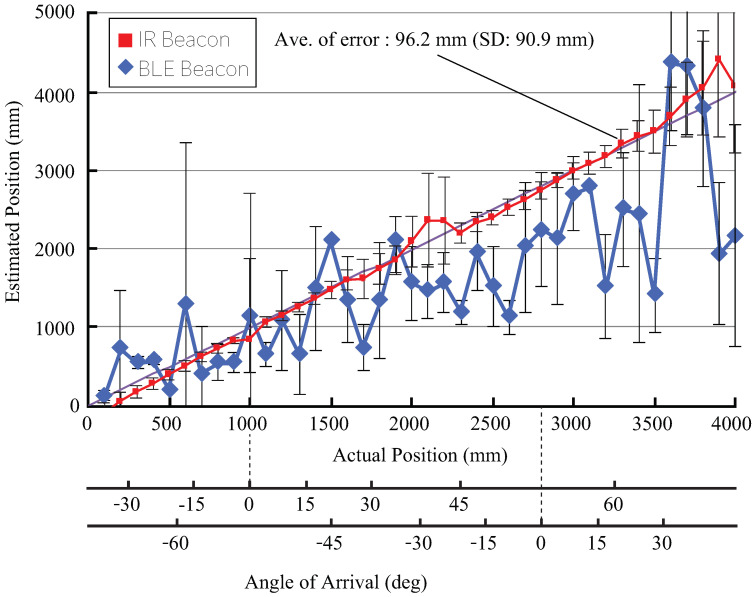
Comparison of positioning performance using a single beacon (the two receivers were installed on the ceiling at distances of 1000 mm and 2800 mm).

In this experiment, two IR receivers (height: 2.6 m) and a dozen of iBeacons (height: 1.52 m) were used for one passage of 5.6 m in length; moreover, the positioning object in the passage was moved by 100 mm to perform positioning for 15 s at each point, and then, the positioning accuracy was compared. In the iBeacon system, the fingerprint of RSSI was measured in a 100 mm interval prior to the experiment, and positioning was performed based on coincidence. The results are shown in Figure 5. From these results, it can be noted that the average error of the iBeacon was 648 mm, while that of the IR beacon was 96.2 mm, and the usefulness of the indoor positioning system using the IR beacon was confirmed.

**Figure 6:**
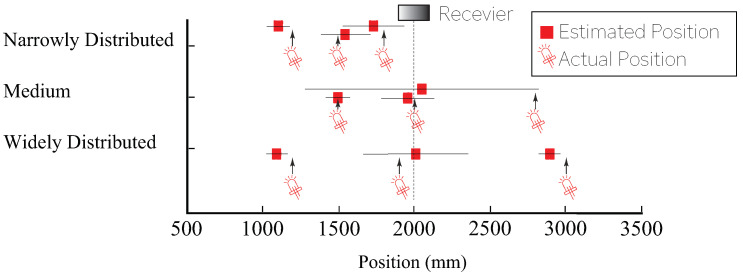
Comparison of positioning performance using multiple beacons (beacons were arranged at 1200, 1900, and 3000 mm distances for a widely distributed configuration, 1500, 2000, and 2800 mm for the medium condition, and 1200, 1500, and 1800 mm for a narrowly distributed configuration, respectively.).

Moreover, in the positioning experiment with multiple IR beacons, a verification experiment on positioning accuracy using three IR beacons was performed on the assumption that multiple IR beacons were present. Moreover, the positioning of the IR beacon was performed in three ways and was performed for 15 s for each situation. The results are shown in Figure 6. The average error was 147 mm, and it was confirmed that effective positioning was possible even in situations in which multiple IR beacons were present.

### 4.3. Positioning in a Dynamic Environment

**Figure 7:**
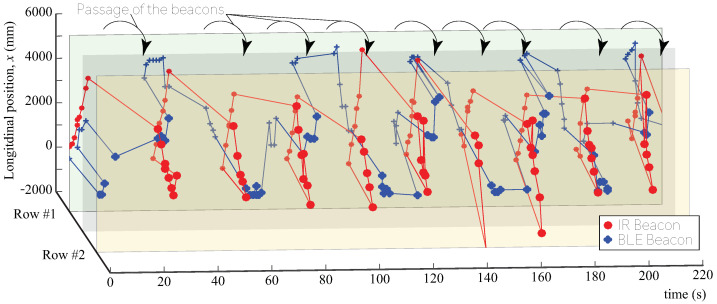
Three-dimensional plot of positioning results. The red line represents the IR beacon, and the blue line represents the BLE beacon. The transverse axis of the graph represents the passage of time; the longitudinal axis represents the depth of the passage (position described as *x* in Figure 4); moreover, the depth axis represents the two passages (transverse axis in Figure 4). While the IR beacon is able to track movement for 10 laps by moving back and forth between two paths, the BLE beacon failed to track the movement correctly.

In the positioning experiment in Case 2, the dynamic measurement was conducted on the assumption that a pedestrian as a measurement object travels through the store. In this measurement experiment, the equipment to be attached to the measurement object was attached to the cart, as shown in Figure 4, and the state of traversing 10 times through the two passages was measured by pushing the cart. The results are shown in Figure 7. From this result, it can be observed that 10 round trips were measured in the positioning by the IR beacon; in contrast, the iBeacon results indicate a measurement of only five round trips. From this result, it can be said that the positioning result of the IR beacon captured the migration behavior more accurately.

### 4.4. Discussion

In both static and dynamic environments, the fingerprint-type indoor positioning method using BLE could not perform accurate positioning even though the number of elements was larger than the proposed method. This is thought to be due to the effects of reflection and absorption by metal shelves and, in some cases, absorption by the human body, because radio waves were used. Furthermore, the attitudes of the devices in the fingerprint learning phase and the verification phase were the same, but when considering actual use, those attitudes did not always match. It is easy to imagine that the performance would be made even worse due to the antenna directivity of the transmitter and receiver.

It is considered that the IR light of each beacon is received by the same receiver when multiple IR beacons are in the same area under the positioning environment. However, the timing of the light emission from each beacon is set to be random in time; consequently, multiple IR beacons are not simultaneously received by the same receiver. In addition, if simultaneous light reception occurs, the data of the sensor value at that time will be lost, but the operation of the positioning system will not be interrupted, and normal data will be acquired again in a state in which each beacon does not overlap temporally or positionally.

In a system in which a receiver and the aggregation server communicate in a one-to-one correspondence, it is assumed that the transmission loss of the data occurs when the distance between the receiver and the aggregation server exceeds the range at which communication is possible in a situation in which positioning is performed over a wide area. To cope with this problem, it is considered that equipment that can relay the communication between the receiver and the aggregation server is required.

The shielding of the IR light of the IR beacon is one of the factors that can result in a positioning error in this system. Therefore, it is desirable to install the receiver on the ceiling of an indoor environment where there is no shield between the receiver and the positioning object to which the IR beacon is attached (i.e., a place with a clear view).

## 5. Implementation in Retail Stores and Measurement of Human Movement

### 5.1. ZigBee Network

The communication system of the ZigBee module, which transmits the sensor value corresponding to the light of the beacon received by the IR receiver to the integration server, was installed in the IR receiver for practical installation in an actual store. When the receiver receives the light transmitted from the IR beacon, the module transmits the time data at that moment, the sensor value, the ID information of the beacon, and the identification number of the communicating module to the aggregation server. Here, the receiver that is installed in the environment that receives the signal of the IR beacon is identified by the correspondence between the individual identification number set in the communication module and the mounted receiver.

When multiple ZigBee modules are used, a communication environment called ad hoc communication is constructed. In the communication between the receiver transmitting the data and the aggregation server in this communication environment, the intermediate receiver can perform the communication as a repeater. On the other hand, true three-dimensional positioning can be also achieved by placing two sensors orthogonally. In this case, the lateral size of the slit should be widened to cover the measurement area. However, the positioning might become less tolerant of reflection.

### 5.2. Measurement of Pedestrian Flow

In this feasibility study, we measured the flow-line data, assuming that certain pedestrians were walking in the retail store.

**Figure 8:**
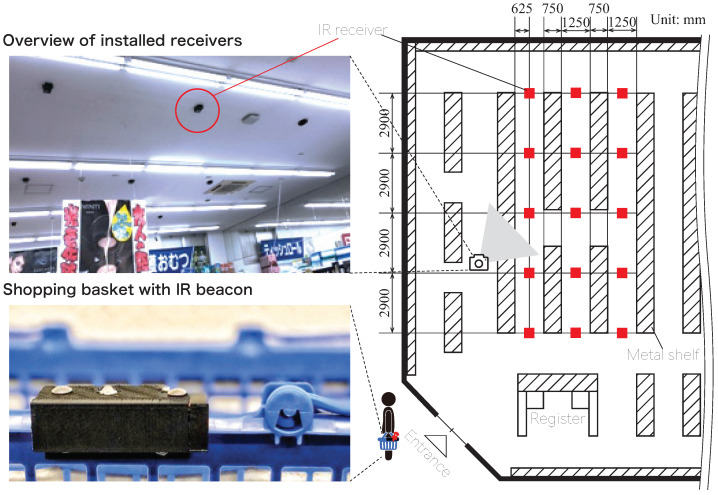
Layout of IR receivers installed in the retail store. The receivers were connected using ZigBee, and power was taken from the outlet. IR receivers were installed on the ceiling of the retail store in the cosmetics sales area (∼40 m${}^{2}$).

This study was conducted in a section of a drug store with IR receivers installed on the ceiling, as shown in Figure 8. Five receivers were attached to the ceiling along each aisle, and the aisle consisted of three rows. The coverage of these receivers was about 40 m${}^{2}$. All receivers were wirelessly connected by ZigBee, and along with the receiver ID, the beacon ID and its AoA information were sent to the server PC via the ZigBee network. Based on this information, the server PC could calculate the position of each beacon in real time.

We also conducted a verification experiment to verify whether the indoor positioning system could measure the transition of the positional relationship between two pedestrians. In this experiment, we measured the behavior of two participants in the store, simulating the behavior of a service employee and customer. The two participants performed the behavior three times according to the five scenarios listed in Table 2, which were considered to be the behaviors related to the service evaluation, obtained from interviews with the salesperson of a drug store.

**Figure 9:**
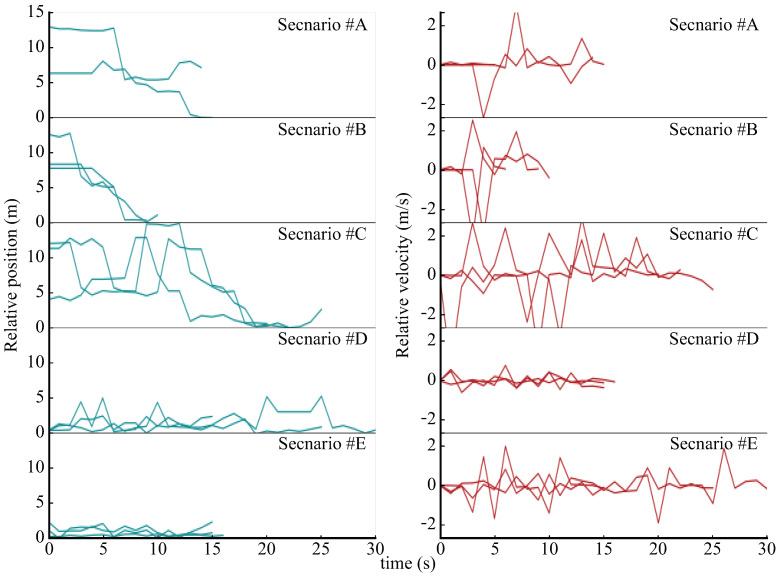
Behavior of the tracked relative relationships among multiple IR Beacons.

Based on this experiment, Figure 9 shows the plots of the relative distance divided into the scenarios of two pedestrians, which were calculated using the obtained log data. There was a difference of approximately several seconds between the relative distances in Scenarios #A and #B before the distance converged to zero. In addition, when compared to Scenario #C, it is possible to observe that the relative distance converged to zero as Scenarios #A and #B monotonously decreased, while they moved away or approached, although they moved differently. Conversely, it was not possible to observe a significant difference between Scenarios #D and #E. However, it can be noted that the distance fluctuated as Scenario #E moved marginally up and down.

Furthermore, focusing on the relative velocity, it was interesting that there was no great difference in the maximum peak value between Scenarios #A and #B, which was different from the initial prediction. It can be observed that, even if an order is used, such as in Scenarios #A and #B, the salesperson will probably move quickly. This point shows that people behave naturally at the service site, unlike machine movements. Moreover, it can be observed that the number of vertical movements in Scenario #C was larger than that of #A and #B. With regard to Scenarios #D and #E, it can be observed that the dispersion of vertical movements was larger in Scenario #E, which suggests that a speed adjustment was made to maintain the distance between the pedestrians while they moved. Additionally, the analysis in [72] was performed using the proposed sensor in the store.

### 5.3. Improvement as a Sensor Network

**Figure 10:**
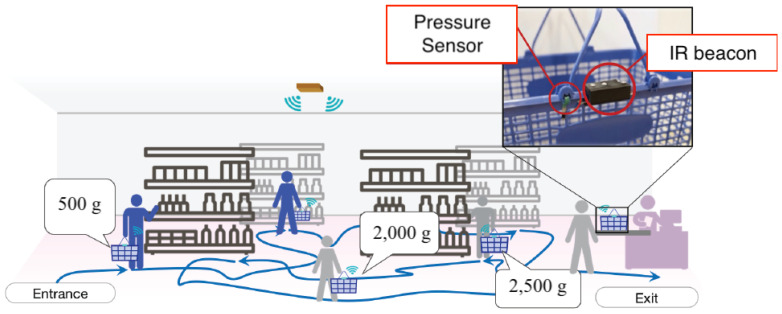
Measuring the interaction between service actors (customer and merchandise) while customers moved in stores [13].

As an example of extending this indoor positioning system to a sensor network, a system to monitor the weight in a shopping cart was constructed [13]. As shown in Figure 10, we attached a pressure sensor to the handle of the shopping basket at the junction of the body of the basket. The pressure sensor changed its resistance value by being compressed between the handle and the body owing to the weight inside the basket. The resistance value of the sensor was transmitted to the receiver by the adjacent IR beacon, and the weight in the shopping basket could be estimated based on the resistance value.

We conducted an experiment to verify the performance of our proposed sensor network system for retail stores. There were two types of shopping baskets: those on wheels that were pushed and those that were carried by hand. The former type could be equipped with a tablet. If baskets were built to certain specifications, it would be relatively easy to apply and thus develop a type of hand-held sensor.

Based on the size of a typical shopping basket, we added loads of approximately 5 kg. To imitate the real-life use of a shopping basket, we loaded items (945 g per item) into the basket and measured the resistance of the pressure sensor. This was conducted every 10 s starting from 1 min after loading to allow the system to stabilize. We measured the resistance 14 times in total, where the weight increased and decreased seven times each. To reduce the influence of the center of gravity, the pressure sensor was installed in a diagonal line. In addition, to produce a more sensitive response to weight, we connected the pressure sensors in series.

**Figure 11:**
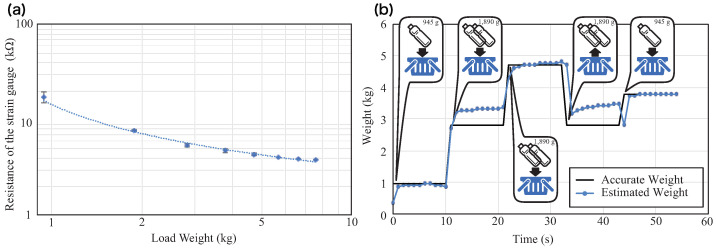
(**a**) Behavior of the strain gauge resistance to loading weight. (**b**) Behavior of estimated weight with changes of the load weight.

In Figure 11a, the horizontal axis shows the weight *w* in the shopping basket, and the vertical axis shows the resistance value *R* indicated by the pressure sensor. It can be observed that the standard error was large while the weight was low, and this became smaller as the weight increased. The standard deviation was approximately 2.2 at approximately 1 kg, but reduced to 0.1 and 0.2 at approximately 2 kg and to less than 0.03 at approximately 5.5 kg.

We estimated the weight of the actual load in the shopping basket using the sensor data, which were sampled from the sensor at 0.1 Hz. The linear prediction model was constructed from the measured data, and an experiment to estimate the weight in the shopping cart was conducted. The items in the shopping basket were changed, and the change in the estimated value was measured. We tracked the sequential behavior that resulted in increases (i.e., placing new items into the basket) and decreases (i.e., returning items to the shelf) in the load weight. The results are shown in Figure 11b. The data were unstable for approximately 10 s after loading the heavy objects, but stabilized thereafter. This was speculated to be due to the characteristics of the pressure sensor. Overall, it was determined that changes in the weight of the basket could be tracked using our proposed system.

### 5.4. Discussion

In this section, we examine the method used to acquire the traffic lines of multiple beacons in the receiving range of the receiver by using a simulation.

**Figure 12:**
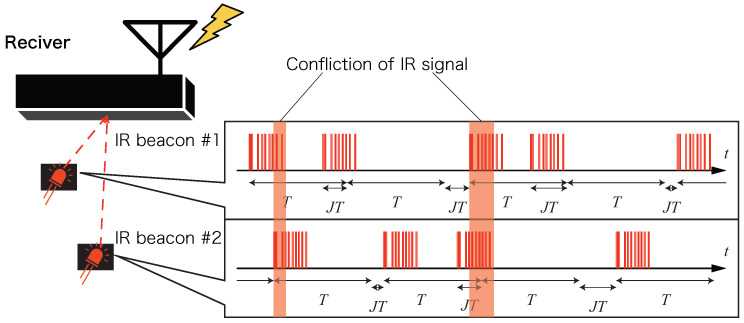
Data loss due to temporal conflict caused by increasing the number of beacons.

**Figure 13:**
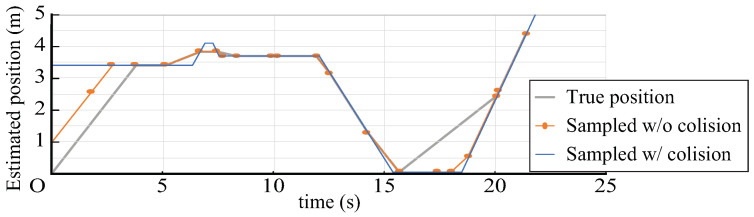
Example of estimated flow lines including collision.

The beacon transmitted its own beacon ID after a random waiting time that was uniformly distributed in the range of ((1−J)T,(1+J)T) before each transmission by using a preset average transmission interval *T* and fluctuation width *J* of the transmission interval. The receiver decoded the beacon ID from the transmitted signal and estimated the position to obtain a sample of the position of the beacon; furthermore, it estimated the flow line by interpolating and extrapolating the positions between the samples. Here, when multiple communications were performed simultaneously because the decoding of the beacon ID and estimation of the position could not be performed, the samples of all beacons transmitted at the time when the collision occurred could not be processed (Figure 12). In this case, it was considered that the error between the estimated flow line and the true flow line was larger than that in the case where no collision had occurred. Figure 13 shows an example of the true trajectory of a beacon, the estimated trajectory if no collision occurred, and the estimated trajectory if a collision occurred and certain samples were missing.

The simulation presented in this section was performed under the following conditions:

- The beacon moved along a path with a length of 5 m. The location of the beacon was expressed in one-dimensional coordinates, with one entrance at a point of 0 m and the other entrance at a point of 5 m.
- The receiver could receive a signal transmitted from a beacon moving in the passage at a time that did not collide with the beacon and obtain a sample of the beacon position without error. All signals transmitted at the time of collision were ignored.
- In the simulation of a single-trajectory estimation, the beacon looped through the next procedure to determine the next destination in the path.
  Step 1 The starting point was 0 m.
  Step 2 The following steps were selected at random:
    - One-eighth of the time, move to the 0 m point, and exit the passage.
    - Move to the 5 m point with a one-eighth chance of getting out of the way.
    - Move randomly to [0, 5] m with a probability of three-quarters; then, rest for a random waiting time of [0, 5] s.
  Step 3 If the path was exited, the simulation would end. Otherwise, the process returned to step 2.
- The beacon selected a moving speed of 80±10 m/min for each movement and performed uniform linear motion at this speed. However, when the selected moving speed was lower than 30 m/min, it was treated as 30 m/min, and when the selected moving speed was higher than 130 m/min, it was treated as 130 m/min.
- When the obtained number of sample points was zero or one in the simulation of a single flow line estimation, it was excluded from the calculation of the root mean squared error (RMSE) to the true value of the estimated flow line.
- The simulation was repeated until each value to be measured was sufficiently converged.

**Figure 14:**
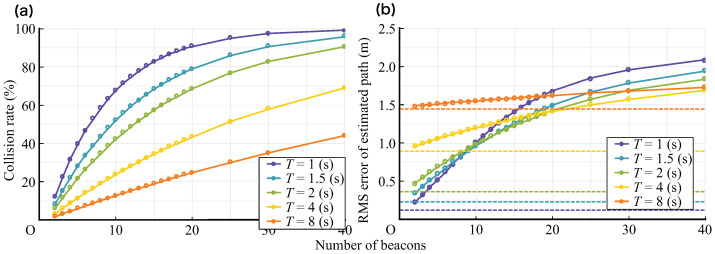
(**a**) Collision rate vs. the number of beacons (J=0.5). (**b**) Root mean squared error (RMSE) of the estimated path vs. the number of beacons (J=0.5). Dashed lines show the RMSE without collision for each *T*.

The collision rate with respect to the number of beacons when the average transmission interval *T* changed is shown in Figure 14a. In any *T*, the collision rate increases as the number of beacons increases. In addition, with the same number of beacons, as the value of *T* increases, the collision rate decreases. Similarly, when the average transmission interval *T* changes, the RMSE of the estimated flow line relative to the number of beacons relative to the true value is shown in Figure 14b.

It can be confirmed that the error tends to increase as the number of beacons increases. Further, in a region in which the number of beacons is small, as the value of *T* decreases, the error decreases; however, in the region in which the number of beacons is large, the relationship is generally reversed. It is assumed that this is because the small interval between samples in the region in which the number of beacons is small and the low collision rate in the region in which the number of beacons is large contributed to the improvement of the accuracy of the flow line estimation. Further, T=4 s and T=8 s show large errors in the region in which the number of beacons is small. It is considered that this is because the flow line generated by the simulation is satisfactory for the average transmission interval and the time length of the flow line is short for the average transmission interval; therefore, the details of the flow line could not be estimated correctly. Thus, it can be said that *T* must be appropriately set according to the size of the passage, the moving speed of the object, and the moving pattern.

**Figure 15:**
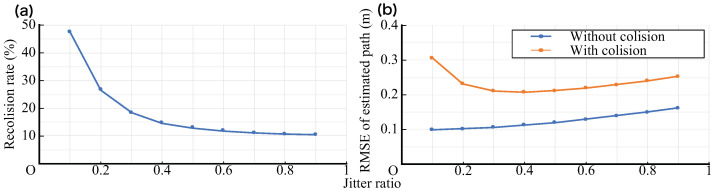
(**a**) Re-collision rate vs. jitter ratio *J* (N=2,T=1 s). (**b**) RMSE of estimated path vs. jitter ratio (N=2,T=1 s).

Figure 14a,b show the long-term frequency of collisions and the accuracy of the trajectory estimates affected by them. While a large number of samples is important in the long term for flow estimation, it is also important from the perspective of data utilization, where the samples are not continuously missing. The likelihood of short-term collisions is considered to depend on the fluctuation width, *J*. For example, if there is no randomness in the latency (J=0), the collision of the first transmission results in the collision of the subsequent transmissions. The rate at which the next transmission of a conflicting transmission will collide again for *J* is shown in Figure 15a. Thus, it was confirmed that as the value of *J* increases, the re-collision rate decreases; in particular, in the region where *J* is small, the decrease in the re-collision rate is more effective.

Similarly, the RMSE for the true value of the estimated flow line for *J* is shown in Figure 15b. It can be confirmed that the error tends to increase as *J* increases, regardless of the presence or absence of collision. It is considered that the accuracy of the flow line estimated by interpolation becomes worse because the samples become unequal because of the increase in *J*. Conversely, in the case of collision, the error decreases when *J* increases in the region where *J* is small. It is considered that this is because the loss of continuous samples is made less likely to occur by increasing *J*. Because of the collision, a loose V-shape is drawn as a whole to obtain the point in which the error reaches its minimum. It is necessary to select the value of *J* at which the error becomes minimal depending on the number of beacons and the value of *T*.

**Figure 16:**
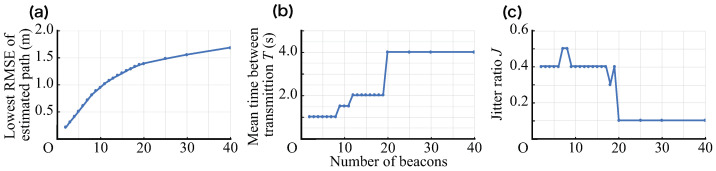
(**a**) Lowest RMSE of the estimation path vs. the number of beacons. (**b**) Mean time between transmissions *T* that achieved the lowest RMSE, which is shown in Figure 16a. (**c**) Jitter ratio *J* that achieved the lowest RMSE, which is shown in Figure 16a.

Finally, while considering the combination of *T* and *J* that can perform the most accurate flow line estimation, the value of the RMSE achieved in T∈{1,1.5,2,4,8}[s], J∈{0.1,0.2,⋯,0.9} performed in this simulation is shown in Figure 16a. The values of *T* and *J* at that time are shown in Figure 16b,c, respectively.

## 6. Conclusions

In this paper, a sensing framework for a VCC process in retail stores was proposed by improving an environment recognition based indoor positioning system with high positioning performance in a metal shelf environment. The conventional indoor positioning systems use radio waves; therefore, errors are caused by reflection, absorption, and interference from metal shelves. An improvement in positioning performance was achieved in the proposed method by using an IR slit and IR light, which avoids such errors. These positioning were calculated under the assumption that the IR beacon is located on a straight line (e.g., the aisle between the shelves) beneath the receiver, whereas the real movements could perform off-the-line as well. The system was designed to recognize many and unspecified people based on the environment recognition method that the receivers had installed, in the service environment. In addition, sensor networking was also conducted by adding a function to transmit payload and identification simultaneously to the beacons that were attached to positioning objects. The effectiveness of the proposed method was verified by installing it not only in an experimental environment with ideal conditions, but posteriorly, the system was tested in real conditions, in a retail store. In our experimental setup, in a comparison with equal element numbers, positioning identification was possible within an error of 96.2 mm in a static environment in contrast to the radio wave based method where an average positioning error of approximately 648 mm was measured using the radio wave based method (Bluetooth low-energy fingerprinting technique). Moreover, when multiple beacons were used simultaneously in our system within the measurement range of one receiver, the appropriate setting of the pulse interval and jitter rate was implemented by simulation. Additionally, it was confirmed that, in a real scenario, it is possible to measure the changes in movement and positional relationships between people. This result shows the feasibility of measuring and evaluating the VCC process in retail stores, although it was difficult to measure the interaction between actors.

In future work, in order to measure the interaction between service actors more accurately, it will be necessary to measure dynamic measurement performance. Moreover, it will be important to combine this approach with other sensors; in particular, a positioning system that obtains its own position by integral calculation and a technology that corrects the estimated position based on its statistical information. In addition, since the light emission timing can be adjusted by bidirectional communication instead of one-way communication, as in this case, positioning efficiency should be improved even when multiple IR beacons are present. However, this leads to increased battery consumption and hardware, so there is a trade-off between the desired accuracy and how complicated the system can be in the usage environment. In the future, we plan to work on a feasibility study of how the quality of service will change by measuring and intervening in the interaction between salespeople and customers at actual stores.

## Figures and Tables

**Table 1:** Comparison of popular indoor positioning methods.

Positioning Method	GPS, IMES [14]	IPS [15]	BLE [16]	PDR [17,18,19]	Camera [20]	Motion Capture [21]	RFID [22,23]	IR Beacon [24,25]
Recognition Type	Self-positioning				Environmental positioning			
Positioning Method	Passive	Active		Passive		Active		
Signal	Radio wave	Visible light	Radio wave	Acceleration, angular velocity, magnetism, atmospheric pressure	Visible light	Infrared light	Radio wave	Infrared light
Localization Method	Code localization	Finger-printing	Lateration, fingerprinting	Strapdown systems	Triangulation	Triangulation	Proximity method	Triangulation
Equipment for Positioning Object	Smartphone				-	Reflection marker	RFID tag	IR beacon
Equipment for Environment	IMES transmitter	Lighting equipment	BLE transmitter	-	Camera	Capture camera	RFID receiver	IR receiver
Error factor	Reflection, absorption	Reflection, absorption	Reflection, absorption	Accumulated error	Shielding	Shielding	Reflection, absorption	Shielding
Installation Cost	-	Low	Low	-	Medium	Low	Medium	Medium
Hardware Cost	Low	Medium	Medium	Medium	Low	High	Medium	Low
Calculating Cost	Medium	Medium	Medium	High	High	Low	Low	Low
Multiple Recognition	Strong				Week		Strong	
Order of Estimation Error	∼10 m	∼3 m	∼3 m	Time increase	∼0.2 m	∼0.1 m	∼5 m	∼1 m
Accuracy in Metal Shelf Environment	Unavailable	Available	Low	Available	High	High	Low	High

**Table 2:** Five configured operational scenarios and physical characteristics.

Scenario	Order to the Participants	Expected Relative Distance	Expected Relative Velocity
#A	Staff approaches the customer slowly at the shortest distance	Decrease slowly and monotonically	The maximum value is large and converges to zero
#B	Staff approaches the customer quickly at the shortest distance	Decrease sharply and monotonically	The maximum value is small and converges to zero
#C	Staff approaches the customer while taking an evasive action	Decrease with variation	Converge to zero with variation
#D	Staff stops at the same point and talks with the customer	Maintain a constant value	Maintain zero with few variations
#E	Staff walks while talking with the customer	Maintain almost a constant value with small fluctuation	Maintain almost zero with small fluctuation
